# Research status and progress on the relationship between obstructive sleep apnea and pulmonary nodules

**DOI:** 10.3389/fmed.2026.1703966

**Published:** 2026-03-25

**Authors:** Ying Fu, Li Xiao

**Affiliations:** Department of Sleep Medicine Center, Shengjing Hospital of China Medical University, Shenyang, China

**Keywords:** chronic inflammatory responses, CPAP, intermittent hypoxia, obstructive sleep apnoea, pulmonary nodules

## Abstract

Obstructive Sleep Apnea (OSA) is a common sleep-disordered breathing. The intermittent hypoxia and chronic inflammation caused by OSA may promote pathological changes in lung tissue and increase the risk of pulmonary nodules. This article systematically reviews the epidemiological evidence, potential mechanistic pathways, diagnostic challenges, and therapeutic implications of the relationship between OSA and pulmonary nodules. Existing observational studies have shown that the incidence of pulmonary nodules in OSA patients is significantly increased, and the severity of OSA is positively correlated with the risk of malignant nodules. Intermittent hypoxia activates oxidative stress and inflammatory response through HIF-1α/NF-κB signaling pathway, which may be the core mechanism of OSA promoting the occurrence and development of pulmonary nodules. Continuous positive airway pressure (CPAP) treatment can reduce the levels of inflammatory factors and may delay the malignant transformation of pulmonary nodules. However, the existing evidence mainly comes from observational studies, with insufficient control of confounding factors, and the causal relationship needs to be further verified.

## 1 Introduction

Obstructive Sleep Apnea (OSA) is a common sleep-disordered breathing characterized by recurrent partial or complete obstruction of the upper airway during sleep, resulting in apnea, hypopnea, and nocturnal awakenings. The pathophysiological mechanism of OSA is mainly related to the abnormal anatomical structure and muscle dysfunction of the upper airway, such as excessive hypertrophy of the pharyngeal soft tissue, hypertrophy of the tongue base and abnormal soft palate structure, which may lead to airway collapse. In addition, inadequate nervous control of the upper airway muscles and decreased sensitivity of the respiratory center to carbon dioxide and oxygen are important causes. Obesity is an important risk factor for OSA, and weight gain leads to fat deposition in the pharyngeal wall, which further aggravates airway compression (1–3).

The impact of OSA on general health is extensive and profound. Chronic intermittent hypoxia and sleep fragmentation not only affect sleep quality, but also may lead to metabolic disorders and cardiovascular diseases. Studies have shown that patients with OSA have a significantly increased risk of hypertension, heart disease and stroke, and are also closely related to metabolic syndrome, diabetes, cognitive impairment and depression (4, 5).

Pulmonary nodules, which appear as round or quasi-round shadows less than 3 cm in diameter on imaging, can be divided into tuberculous nodules, granulomatous nodules and neoplastic nodules. Based on density and edge characteristics, pulmonary nodules can be divided into solid nodules, part-solid nodules, and pure ground glass nodules, among which part-solid nodules have the highest malignant probability (6, 7). Low-dose spiral CT scan is an important method for the diagnosis of pulmonary nodules, and the detection sensitivity can reach 95%. Smoking, air pollution, occupational exposure and certain microbial infections are the main risk factors for pulmonary nodules (8, 9).

In recent years, studies have found that OSA may be closely related to the occurrence of a variety of lung diseases, especially the formation mechanism of pulmonary nodules. Intermittent hypoxia and chronic inflammation caused by OSA may promote pathological changes in lung tissue, thereby increasing the risk of pulmonary nodules. Focusing on the four major themes of epidemiological evidence, mechanistic pathways, diagnostic challenges and therapeutic significance, this article systematically reviews the research status and progress of the relationship between OSA and pulmonary nodules, aiming to provide a theoretical basis for clinicians to pay attention to pulmonary nodule screening and follow-up when managing patients with OSA.

## 2 Epidemiological evidence: the association between OSA and the risk of pulmonary nodules

### 2.1 Summary of evidence from observational studies

Multiple observational studies have indicated an association between OSA and the risks of pulmonary nodules and lung cancer. The results of Yao et al. (10) meta-analysis suggest that the risk of lung cancer in OSA patients increases. Ding et al. (11) retrospective study showed that the incidence of isolated pulmonary nodules in OSA patients was significantly higher than that in the control group, and the severity of OSA was positively correlated with the incidence of nodules. Chen et al. (12) research further found that the serum carcinoembryonic antigen (CEA) level in OSA patients was significantly higher than that in the non-OSA control group, and the severity of OSA was positively correlated with the CEA level, suggesting that OSA may increase the malignant risk of pulmonary nodules by promoting the release of tumor-associated antigens. In addition, OSA patients often have other nodular lesions such as thyroid nodules, and this comorbidity state may exacerbate the overall health risks of the patients (11).

### 2.2 Special groups: patients with overlap syndrome

The Overlap of OSA and chronic obstructive pulmonary disease (COPD) is called Overlap Syndrome (OS), and its prevalence is between 10 and 15%. OSA patients with COPD have a higher prevalence of pulmonary hypertension, which may be because such patients have more severe hypoxemia during sleep, which is prone to cause pulmonary artery constriction (13, 14). Due to the coexistence of upper airway obstruction and lower airway obstruction, patients with OS have more severe hypoxia at night, and the risk of pulmonary nodules and malignant transformation may be further increased, which deserves special attention in clinical practice (Table 1).

**Table 1:** Key clinical studies linking OSA and pulmonary nodules.

Author/Year	Methods	Objects of study	Main findings	Effect size/Conclusion
Ding et al. (2024) (11)	Retrospective study	OSA patients vs. control group	The incidence of solitary pulmonary nodules is significantly increased in OSA patients	The incidence of isolated pulmonary nodules is positively correlated with the severity of OSA.
Chen et al. (2023) (12)	Case–control study	OSA patient cohort	The serum CEA level of OSA patients was significantly elevated	The increase of serum CEA level is positively correlated with the severity of OSA
Huang et al. (2023) (64)	Cohort Study	CPAP treatment VS no treatment	CPAP treatment is associated with a reduced incidence of lung cancer.	HR = 0.79 (95%CI:0.65–0.96)
Study on OS (2018–2023)	Observational study	Patients with OSA and COPD	The risk of pulmonary nodules in patients with OS is further increased.	Patients with OS experience more severe hypoxia at night and require special attention.

COPD, chronic obstructive pulmonary disease; CPAP, continuous positive airway pressure; OS, overlap syndrome; HR, hzard risk; CI, confidence interval.

## 3 Epidemiological evidence: the association between OSA and the risk of pulmonary nodules

OSA leads to frequent hypoxemia and reperfusion at night, which induces oxidative stress. The pathological changes induced by periodic hypoxia may be closely related to the formation of pulmonary nodules. The potential mechanisms of OSA in promoting the occurrence and development of pulmonary nodules are described below from the molecular and cellular levels.

### 3.1 Intermittent hypoxia and oxidative stress

#### 3.1.1 Nicotinamide adenine dinucleotide phosphate(NADPH) oxidase activation pathway

The NADPH oxidase (NOX) is a key enzyme system that induces Reactive Oxygen Species (ROS) production under intermittent hypoxia. Chronic intermittent hypoxia significantly upregulates the expression levels of NOX2 and NOX4 in vascular endothelial cells, smooth muscle cells, and circulating white blood cells (15). Intermittent hypoxia amplifies the redox imbalance by activating the sympathetic nerve and angiotensin II signaling pathways. The NADPH oxidase catalyzes the transfer of electrons from NADPH to molecular oxygen, generating superoxide anion (O₂^−^), which then converts to reactive oxygen species such as hydrogen peroxide (H₂O₂). Studies have shown that ROS derived from NADPH oxidase plays a central role in the sensitization of pulmonary vagal nerve C fibers induced by intermittent hypoxia. The application of NADPH oxidase inhibitors (such as apocynin) or superoxide dismutase mimetics can significantly improve airway hyperreactivity induced by intermittent hypoxia (16).

#### 3.1.2 Mitochondrial dysfunction and ROS production

Mitochondria are the main source of ROS production under intermittent hypoxia conditions. Each respiratory pause event after reoxygenation leads to a brief hyperpolarization of mitochondria, resulting in the accumulation of superoxide anions and hydrogen peroxide (17). Persistent chronic intermittent hypoxia alters mitochondrial dynamics (fusion/fission balance), impairs the efficiency of oxidative phosphorylation, and damages mitochondrial DNA, thereby continuously generating ROS. The level of mitochondrial DNA in peripheral blood of OSA patients is decreased, which is a sign of mitochondrial damage (18). Dysfunction of mitochondrial electron transport chain complex I is particularly crucial in ROS production induced by intermittent hypoxia.

#### 3.1.3 Xanthine oxidase system

The xanthine oxidoreductase system significantly contributes to ROS production during hypoxia-reoxygenation. Under hypoxic conditions, xanthine dehydrogenase is converted to xanthine oxidase, which metabolizes hypoxanthine to uric acid during reoxygenation, while producing superoxide anion and hydrogen peroxide as by-products (19). Serum uric acid levels are elevated in OSA patients, which can be used as an indirect marker of xanthine oxidase activation and oxidative stress load.

#### 3.1.4 Oxidative stress-mediated DNA damage

Excessive ROS induces DNA damage and promotes malignant transformation of pulmonary nodules through multiple mechanisms. Reactive oxygen species directly attack DNA molecules to form oxidative damage markers such as 8-hydroxy-deoxyguanosine (8-OHdG), leading to base mismatch and mutation accumulation (20). Oxidative stress can also induce ER stress, leading to the accumulation of unfolded or misfolded proteins, further activating apoptotic pathways or promoting malignant transformation of cells. In addition, ROS produces aldehydes such as malondialdehyde (MDA) through lipid peroxidation, which damages cell membranes, lipoproteins and other lipid molecules. MDA levels are significantly increased in OSA patients, which can be used as a potential biomarker of oxidative stress (21).

### 3.2 Systemic inflammation and immune microenvironment changes

#### 3.2.1 Tumor-associated macrophages are polarized

Among tumor immune effector cells, macrophages are the main regulators in the tumor microenvironment. Intermittent hypoxia and sleep deprivation can promote the recruitment of macrophages to the tumor site, and change the polarity of macrophages through a hypoxia inducible factor-1α(HIF-1α) dependent mechanism (22). Normal macrophages (M1 type) have anti-tumor activity, whereas tumor-associated macrophages (M2 type) promote tumor growth, invasion, and metastasis. Intermittent hypoxia promotes the polarization of macrophages to M2 phenotype by activating HIF-1α signaling pathway, releasing immunosuppressive factors such as IL-10 and TGF-β, and inhibiting the production of pro-inflammatory factors, thus creating an immune microenvironment conducive to tumor progression.

#### 3.2.2 Recruitment and activation of myeloid-derived suppressor cells (MDSC)

Myeloid-derived suppressor cells (MDSC) are a group of immature myeloid cells with strong immunosuppressive function, which play a key role in tumor immune escape. Hypoxic tumor microenvironment induces the expression of ENTPD2 (also known as CD39L1) through HIF-1α, which promotes the migration of MDSC to the tumor microenvironment (23). MDSC suppress anti-tumor immune response through multiple mechanisms:

1) production of ROS (via NADPH oxidase 2) reduces T cell CD3ζ chain expression and inhibits T cell proliferation;
2) Induction of nitric oxide synthase (iNOS) and arginase 1 (ARG1) expression, depletion of L-arginine, leading to anergy of T cells;
3) The secretion of IL-10 and TGF-β induced the differentiation of regulatory T cells (Treg) and the generation of M2 macrophages;
4) Adenosine produced by exonucleases CD39 and CD73 inhibits Zap70, ERK and Akt phosphorylation of T cells and reduces the cytotoxic function of CD8 + T cells (24, 25).

#### 3.2.3 Expansion of regulatory T cells (Tregs)

Regulatory T cells (Tregs) are a key cell population to maintain immune tolerance and suppress autoimmune responses, but they promote immune escape in the tumor microenvironment. Intermittent hypoxia affects the differentiation and function of Tregs through HIF-1αsignaling pathway. HIF-1αis hydroxylated by prolyl hydroxylase (PHDs) under normoxia, then recognized by VHL protein and modified by K48 polyubiquitination mediated by E3 ubiquitin ligase complex, and finally degraded by 26S proteasome (26). In hypoxia, unhydroxylated HIF-1α accumulates and binds to the beta subunit, activating transcription of a variety of metabolic and immune-related genes.

HIF-1α promotes Tregs-mediated immunosuppression through multiple mechanisms. Firstly, HIF-1α directly binds to the FOXP3 gene promoter region to enhance the expression of Tregs’ signature transcription factors. Secondly, HIF-1α induces Treg to secrete inhibitory cytokines such as IL-10 and TGF-β. In addition, HIF-1α promotes the expression of immune checkpoint molecules such as CTLA-4 and PD-1 on the surface of Tregs, enhancing their inhibitory effect on effector T cells (27).

#### 3.2.4 Inhibition of cytotoxic T lymphocyte (CTL) function

The combination of intermittent hypoxia and the accumulation of immunosuppressive cells leads to a significant suppression of cytotoxic T lymphocyte function. ROS and NO produced by MDSC directly damage the TCRζ chain and effector molecules of CTL, reducing their ability to recognize and kill tumor cells. Adenosine in the tumor microenvironment inhibits CTL proliferation, cytokine production, and cytotoxic activity through A2A receptors. In addition, tumor cells and suppressor immune cells highly express PD-L1, which binds to PD-1 on the surface of CTL and induces T cell exhaustion, forming a vicious cycle (28).

### 3.3 Circadian rhythm disruption and sleep fragmentation

#### 3.3.1 Disruption of core circadian genes

Circadian rhythms are precisely regulated by a core transcription-translation feedback loop, with key genes including CLOCK (circadian motor output cycle failure), BMAL1 (brain and muscle ARNt-like protein 1), PER1/2/3 (cyclin), and CRY1/2 (cryptochrome). Studies have shown that the expression patterns of these core circadian genes are significantly altered in OSA patients (29). Yang et al. found that the daily expression pattern of CLOCK, BMAL1 and CRY2 genes was completely abolished in OSA patients, and only PER1 was not significantly down-regulated at midnight (30). Disruption of these core circadian genes may increase genomic instability by affecting the expression of downstream cell cycle regulatory genes and DNA repair genes.

#### 3.3.2 Interaction between HIF-1α and circadian rhythms

There is a close molecular interaction between circadian rhythm disorder and hypoxia signaling pathway. As a key regulator of oxygen metabolism, HIF-1α plays an important role in the regulation of circadian gene expression (31). On the one hand, HIF-1α can directly bind to and activate the promoters of some circadian genes. On the other hand, hypoxia-induced oxidative stress can affect the post-translational modification and stability of core clock proteins. Studies have shown that intermittent hypoxia and continuous hypoxia activate HIF-1α through different pathways, and intermittent hypoxia reduces the stability of HIF-1α, which in turn activates the nuclear factor kappa-B(NF-κB) pathway (32).

#### 3.3.3 Effects of circadian disruption on cancer-related genes

Disruption of sleep and circadian stability is associated with epigenetic modifications of multiple key circadian genes, which in turn alter transcriptional regulation and affect the expression of cancer-related susceptibility genes, while disrupting gene networks that coordinate cell division and DNA repair (33). As a core transcription factor, BMAL1 regulates the expression of cell cycle checkpoint genes (such as p53, Cyclin D1) and DNA damage repair genes. Circadian rhythm disruption leads to abnormal BMAL1 function, which may make cell cycle checkpoints out of control, DNA damage accumulation, and ultimately promote malignant transformation. Continuous Positive Airway Pressure (CPAP) treatment can improve the disorder of circadian rhythm genes to a certain extent, and studies have found that even short-term effective continuous positive airway pressure treatment may be able to repair the disorder of circadian rhythm signaling pathways in patients with obstructive sleep apnea (34).

### 3.4 Key signaling pathways: HIF-1α/NF-κB axis

#### 3.4.1 Synergistic activation of the HIF-1α/NF-κB signaling axis

HIF-1α and NF-κB form a positive feedback loop under intermittent hypoxia. On the one hand, HIF-1α transcriptionally activates multiple NF-κB target genes; On the other hand, NF-κB enhances the gene transcription and protein stability of HIF-1*α* (35). This synergistic activation leads to sustained high expression of proinflammatory cytokines (IL-6, IL-8, TNF-α), chemokines, and vascular endothelial growth factor (VEGF), creating an inflammatory microenvironment conducive to tumor growth. Mice exposed to intermittent hypoxia had an increased incidence of lung metastases (approximately three times that of normal hypoxic mice), an effect attributed to increased hypoxia-vascular endothelial growth factor-A (VEGF-A) expression in the primary tumor, leading to accelerated angiogenesis and blood perfusion, promoting tumor cell intravasation and blood dissemination (36).

#### 3.4.2 Induction of epithelial-mesenchymal transition (EMT)

HIF-1α is a key transcription factor that induces epithelial-mesenchymal transition (EMT), a core process of tumor invasion and metastasis. Intermittent hypoxia up-regulated the expression of EMT-related transcription factors (Snail, Slug, Twist, ZEB1/2) and inhibited the epithelial marker E-cadherin through HIF-1α. It also induced the expression of mesenchymal markers N-cadherin and Vimentin (37).

Studies have shown that chronic intermittent hypoxia enhances the stemness, proliferation, migration and invasion of non-small cell lung cancer (NSCLC) through the ESM1 (endothelial cell specific molecule-1) /HIF-1α pathway (38). ESM1 is overexpressed in a variety of tumors, and intermittent hypoxia significantly promotes ESM1 expression, while ESM1 gene silencing can reverse intermittent hypoxia-induced tumor growth, drug resistance and metastasis.

#### 3.4.3 Cancer stem cell (CSC) maintenance and expansion

Cancer stem cells are the root cause of tumor recurrence and metastasis. Intermittent hypoxia maintains and expands the cancer stem cell population through HIF-1α. HIF-1α directly binds to and activates the gene promoters of tumor stem cell markers (CD44, CD133, OCT4, SOX2) to maintain the self-renewal and multi-directional differentiation ability of cancer stem cells (39). Chronic intermittent hypoxia enhances the stemness of lung cancer stem cells (LCSC) and promotes their resistance to cisplatin and other chemotherapy drugs. Under intermittent hypoxia, cancer stem cells preferentially locate in hypoxic areas and form a cell reservoir that is resistant to treatment (40).

#### 3.4.4 Angiogenesis and tumor microenvironment remodeling

HIF-1αis a major regulator of angiogenesis and promotes tumor neovascularization through transcriptional activation of VEGF, PDGF, ANGPT and other pro-angiogenic factors. The high expression of VEGF-A induced by intermittent hypoxia not only promotes angiogenesis, but also increases vascular permeability, facilitating the entry of tumor cells into the circulatory system (41). In addition, HIF-1αinduces the expression of matrix metalloproteinases (MMPs), degrades the extracellular matrix, and promotes tumor cell invasion. HIF-1α also regulates the activation of cancer-associated fibroblasts (CAF) to further reshape the tumor microenvironment (42).

### 3.5 Sympathetic activation and nerve-tumor interaction

Patients with OSA have recurrent apnea and hypopnea during the night, resulting in persistent activation of the sympathetic nervous system. Sympathetic nerves release catecholamine neurotransmitters (norepinephrine, epinephrine) and activate β-adrenergic receptors (β-AR) on the surface of tumor cells and tumor-related immune cells, forming a neuro-endocrine-immune network that promotes tumor progression.

#### 3.5.1 Chronic stress and β-adrenergic signaling

Experimental and epidemiological evidence suggests that systemic physiological stress response pathways may shape the tumor microenvironment to promote metastasis. These pathways release catecholamine neurotransmitters through the peripheral sympathetic nervous system and stimulate β-adrenergic receptors on the surface of tumor cells and tumor-associated macrophages (43). Experimental studies have found that chronic stress accelerates breast cancer metastasis through the β-adrenergic signaling pathway, which recruits alternative activated macrophages to the primary tumor site. Pharmacological activation of β-adrenergic signaling increases primary tumor metastasis, whereas β-blockers such as propranolol completely inhibit stress-enhanced metastasis (44).

#### 3.5.2 Direct effects of β-AR signaling on tumor cells

β-AR belongs to the G protein-coupled receptor family. After activation, it activates adenylyl cyclase through Gs protein, which increases the intracellular cAMP level, and then activates protein kinase A (PKA) and exchange protein (Epac). These signaling molecules promote tumor progression through multiple mechanisms:

1) Activation of MAPK/ERK and PI3K/Akt signaling pathways to promote tumor cell proliferation and survival;
2) Up-regulating the expression of VEGF and MMPs, promoting angiogenesis and invasion;
3) Inhibit the activity of apoptotic proteins (such as Bad and Bax) and enhance the anti-apoptotic ability of tumor cells (45).

#### 3.5.3 Sympathetic regulation of the immune microenvironment

Sympathetic signaling affects tumor immune response by regulating the function of immune cells. Catecholamines can inhibit the cytotoxic activity of natural killer (NK) cells and reduce their killing ability to tumor cells. In addition, β-AR signaling promotes the polarization of macrophages to the M2 phenotype and enhances their immunosuppressive function. In the tumor microenvironment, the density of sympathetic nerve fibers is positively correlated with the accumulation of MDSC and Tregs, and negatively correlated with the infiltration of cytotoxic T lymphocytes (46).

#### 3.5.4 Potential antitumor effects of β-receptor blockers

Based on the role of sympathetic activation in tumor progression, β-receptor blockers may have anti-tumor potential. Clinical studies have found that cancer patients treated with β-receptor blockers have improved prognosis, especially in breast cancer patients, where β-receptor blockers use is associated with reduced recurrence and mortality (47). For patients with OSA and pulmonary nodules, β-receptor blockers may play an auxiliary anti-tumor effect by blocking the excessive activation of sympathetic nerve induced by chronic intermittent hypoxia, which is worthy of further clinical research.

### 3.6 An integrated mechanism model of OSA promoting the occurrence and development of pulmonary nodules

#### 3.6.1 Core drive: the cascade effects of intermittent hypoxia

Intermittent hypoxia, as the core pathophysiological feature of OSA, is the initiating factor of the whole mechanism network. Intermittent hypoxia initiates downstream cascades through three main pathways: (1) activation of NADPH oxidase and mitochondrial dysfunction, production of excessive ROS, induction of oxidative stress and DNA damage; (2) stabilizing HIF-1α protein and activating transcription of hypoxia-responsive genes; (3) Activation of NF-κB signaling pathway and induction of pro-inflammatory cytokines expression. These three pathways cross each other and form a positive feedback loop to amplify pathological signals (48).

#### 3.6.2 Central integration: the hub of HIF-1α/NF-κB signaling

HIF-1α and NF-κB constitute the central integration node of the mechanistic network. HIF-1α not only directly regulates pro-angiogenic and metabolic reprogramming genes such as VEGF and GLUT1, but also forms a transcription complex with NF-κB to synergistically activate downstream target genes. NF-κB further activates the JAK/STAT3 signaling pathway by inducing inflammatory factors such as IL-6 and TNF-*α* to promote tumor cell proliferation, survival and immune escape. HIF-1α/NF-κB axis also induces the expression of EMT-related transcription factors and promotes the invasion and metastasis of tumor cells.

#### 3.6.3 Remodeling of the microenvironment: establishment of the immunosuppressive network

Intermittent hypoxia and inflammatory signals together reshape the microenvironment of pulmonary nodules and establish an immunosuppressive state. HIF-1α induces the polarization of macrophages to M2 phenotype, promotes the recruitment of MDSC to the tumor site and enhances its immunosuppressive function, and amplifies the Tregs. By producing inhibitory molecules such as ROS, NO and adenosine, depleting L-arginine and secreting IL-10 and TGF-β,these immunosuppressive cells inhibit the function of cytotoxic T lymphocytes at multiple levels and enable tumor cells to escape immune surveillance (49).

#### 3.6.4 Malignant transformation: progression from a nodule to an invasive carcinoma

In the microenvironment of continuous oxidative stress, inflammation and immunosuppression, pulmonary nodules undergo an evolution process from benign to malignant. Oxidative stress-induced DNA damage leads to mutation of key tumor suppressor genes such as p53 and activation of proto-oncogenes. Hif-1α-mediated EMT enables tumor cells to acquire a mesenchymal phenotype and enhance their migration and invasion abilities. The maintenance and expansion of cancer stem cells provide a cell source for tumor recurrence and metastasis. The continuous expression of angiogenic factors promotes neovascularization to provide nutrients and oxygen for tumor growth, while providing a channel for distant metastasis of tumor cells.

#### 3.6.5 Targets for therapeutic intervention

**Figure 1:**
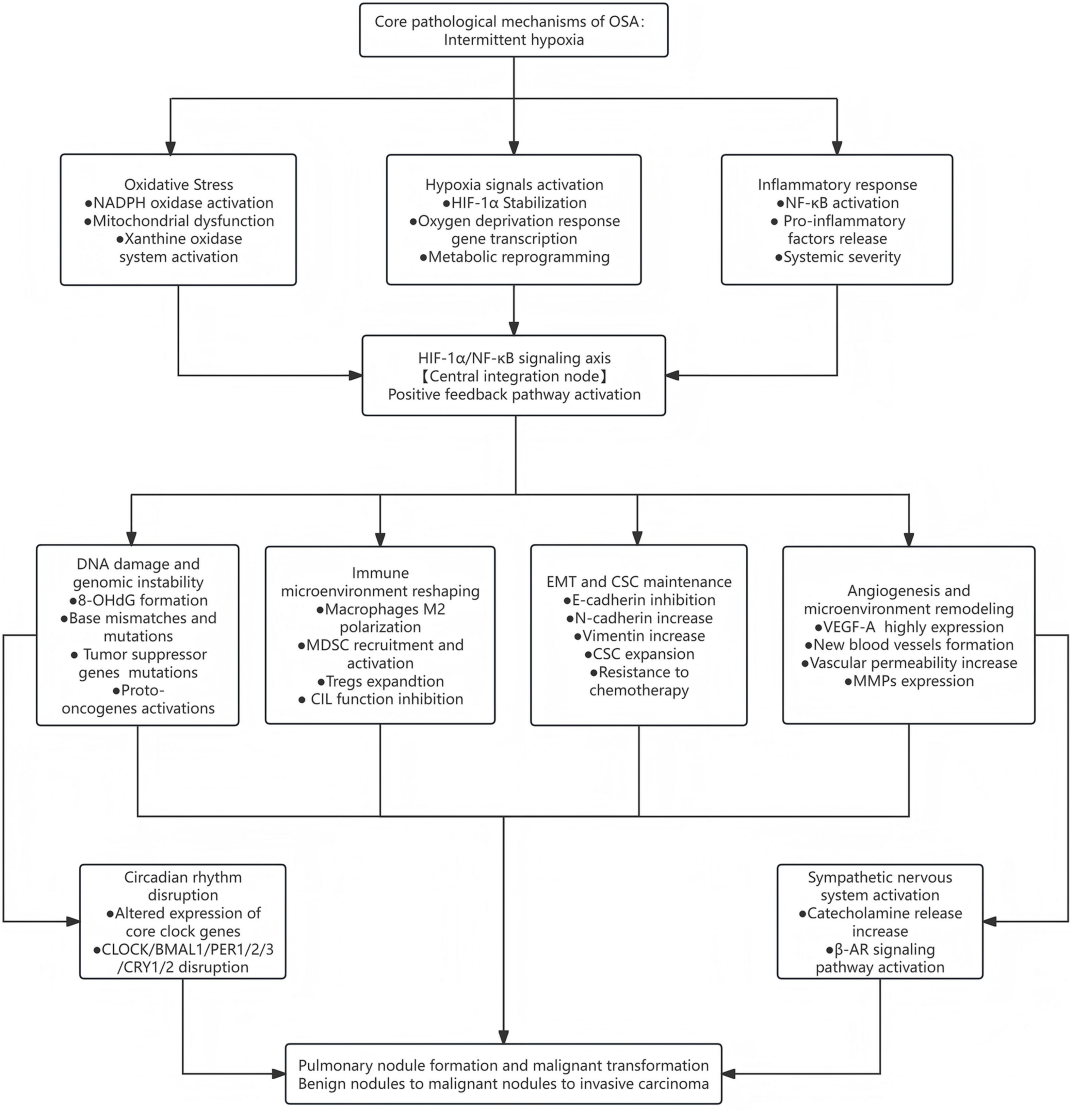
Mechanism pathway map of OSA promoting the occurrence and development of pulmonary nodules.

Based on the above integrated mechanism model, the therapeutic intervention of OSA patients with pulmonary nodules can be started from multiple levels:

1) Source control: CPAP treatment to correct intermittent hypoxia and block the initiating factors of the mechanism network;
2) Antioxidant therapy: NADPH oxidase inhibitors or antioxidants to reduce oxidative stress injury;
3) Anti-inflammatory therapy: NF-κB or JAK/STAT pathway inhibitors to control inflammatory response;
4) Immune regulation: immune checkpoint inhibitors or MDSC targeted therapy restore anti-tumor immune response;
5) β-receptor blockade: blocking the tumor-promoting effect of excessive activation of sympathetic nerve. Multi-target combined intervention may provide a more comprehensive treatment strategy for OSA patients with pulmonary nodules (Figure 1).

## 4 Diagnostic challenge: assessment of pulmonary nodules in patients with OSA

### 4.1 Differences in imaging features

In clinical practice, pulmonary nodules in OSA patients exhibit some specific features. Patients with OSA often show a higher incidence of pulmonary nodules when receiving low-dose computed tomography (LDCT) (50). The imaging characteristics of pulmonary nodules include diameter, edge characteristics, density type and growth rate, etc. These characteristics may be different in OSA patients. Studies have shown that the growth rate of nodules is an important indicator for assessing the risk of malignancy, and the chronic inflammatory state associated with OSA may affect the growth pattern of nodules (51).

### 4.2 Application and limitations of tumor markers

Patients with OSA often show higher levels of tumor markers, which may be related to their chronic hypoxia state and related metabolic syndrome. Carcinoembryonic antigen (CEA) is one of the most commonly used tumor markers in the diagnosis of pulmonary nodules, but its application in patients with OSA has special considerations. Currently, CEA is more commonly used for the diagnosis of adenocarcinoma in non-small cell lung cancer, but the cut-off values vary widely among different studies. CEA is a common biomarker whose level is upregulated in a variety of malignancies. Despite its wide range, it is still valuable in assisting the diagnosis of lung cancer. Since CEA is usually produced during fetal development and stops before birth, it is usually not present in the blood of healthy adults. Currently, CEA is more commonly used for adenocarcinoma diagnosis of non-small cell lung cancer, but its threshold varies widely among studies (52, 53). One Chinese study found that serum CEA levels in non-small cell lung cancer cases were significantly higher than those in benign lung tumor cases and healthy people (13), while another study found that CEA levels in lung adenocarcinoma group were significantly higher than those in lung SCC group and small cell lung cancer group.

Although the above results indicate that CEA is important for clinical diagnosis of lung cancer, however, its role in early diagnosis is less convincing because of poor specificity and the degree of elevation in the early stages is not obvious (54). It is worth noting that about 15% of patients with pneumonia and tuberculosis have mild elevation of CEA (5–10 ng/mL), and the baseline CEA level of smokers is significantly higher than that of non-smokers (*p* < 0.01) (55).

The combination of tumor markers has higher diagnostic value. Studies have shown that concur with those of others which indicate that CEA, CYFRA21-1, SCC-Ag, and pro-GRP play an important role in the differential diagnosis of lung Studies have shown that CEA, CYFRA21-1, SCC-Ag and pro-GRP play an important role in the differential diagnosis of lung cancer. The serum levels of CEA, CYFRA21-1, SCC-Ag and pro-GRP in lung cancer patients were significantly higher than those in benign lung tumor patients and healthy people (*p* < 0.05), indicating that they have certain significance in the diagnosis of lung cancer (54). When interpreting tumor markers in OSA patients, the effects of factors such as smoking history and inflammatory state on baseline levels should be fully considered.

The chronic hypoxia state of OSA patients can affect the specificity of tumor markers through a variety of mechanisms.

Firstly, chronic intermittent hypoxia induces systemic inflammation by activating the HIF-1*α*/NF-κB signaling pathway, resulting in a sustained increase in the levels of proinflammatory cytokines such as IL-6, TNF-α, and CRP (56). These inflammatory mediators can directly stimulate normal epithelial cells to express and release tumor associated antigens such as CEA, resulting in false positive results. Studies have shown that about 15–30% of patients with benign lung diseases such as pneumonia, tuberculosis, and chronic obstructive pulmonary disease have mild to moderate increases in CEA, and the mechanism is closely related to the proliferation of bronchial epithelial cells induced by inflammation and the up-regulation of CEA expression (57). Due to long-term exposure to intermittent hypoxia, the systemic inflammatory state of OSA patients is similar to that of the above diseases, which may lead to a false increase in the baseline level of CEA, thereby reducing the specificity of this marker in the differentiation of benign and malignant nodules.

Secondly, the oxidative stress response induced by intermittent hypoxia is another important mechanism that reduces the specificity of biomarkers. The repeated episodes of hypoxia-reoxygenation during sleep in patients with OSA promote the massive production of reactive oxygen species (ROS), leading to lipid peroxidation, protein oxidation, and DNA damage (15). Oxidative stress can damage the structure of cell membranes, causing the release of cellular contents (including glycoproteins such as CEA) into the blood. Moreover, oxidative stress can further increase the transcription of the CEA gene by activating the MAPK/ERK signaling pathway, thereby further elevating serum CEA levels (58). This non-tumoral increase in CEA levels is particularly significant in OSA patients. Studies have found that the serum CEA levels of patients with severe OSA (Apnea-Hypopnea Index, AHI > 30) are approximately 20–40% higher than those of the control group, and are positively correlated with the severity of hypoxia (12).

Thirdly, OSA-related metabolic disorders can also interfere with the interpretation of tumor markers. Patients with OSA are often complicated with obesity, insulin resistance and type 2 diabetes mellitus, and these abnormal metabolic states form a vicious cycle with chronic inflammation and oxidative stress (1). Obesity itself can stimulate CEA expression through inflammatory factors (such as leptin and resistin) secreted by adipose tissue, while hyperinsulinemia in the state of insulin resistance can promote cell proliferation and CEA release by activating PI3K/Akt signaling pathway (59). Therefore, in patients with OSA and metabolic syndrome, elevated CEA may reflect metabolic disorders rather than malignant lesions, further reducing the diagnostic specificity of this marker.

Finally, the disruption of circadian rhythm in OSA patients may also affect the detection accuracy of tumor markers. Studies have shown that the expression of glycoproteins such as CEA has circadian fluctuation, and the rhythm disorder of OSA patients may lead to a mismatch between the marker detection time point and the normal reference range, increasing the risk of false positive.

In conclusion, chronic hypoxia in OSA patients affects the specificity of tumor markers such as CEA through multiple mechanisms, including inflammatory activation, oxidative stress, metabolic disorders, and circadian dysrhythmia. In clinical practice, the influence of these non-neoplastic factors should be fully considered when interpreting the results of tumor markers in OSA patients. It is recommended to adopt a dynamic monitoring strategy (such as repeated detection at 4–6 weeks intervals) to observe the change trend of marker levels rather than relying on single detection results. For OSA patients with mild elevation of CEA (5–10 ng/mL), comprehensive evaluation should be combined with imaging features, smoking history, inflammatory indicators, etc. If necessary, CPAP treatment should be reexamined to exclude hypooxia-related false elevation (60).

### 4.3 Special considerations in differential diagnosis

Multiple factors should be considered in the differential diagnosis of pulmonary nodules in OSA patients. First, OSA-related chronic inflammation may cause benign nodules to resemble malignant nodules on imaging, increasing the difficulty of differential diagnosis. Secondly, OSA patients are often combined with other pulmonary diseases (such as COPD and pulmonary hypertension), and the imaging changes of these diseases may overlap with pulmonary nodules (61). In addition, with the development of artificial intelligence technology, computer-aided detection systems have shown good prospects in the identification and classification of pulmonary nodules, which may help to improve the accuracy of pulmonary nodule diagnosis in OSA patients (62, 63).

## 5 Therapeutic implications: effect of OSA intervention on the prognosis of pulmonary nodules

### 5.1 Potential protective effects of CPAP therapy

The first-line treatment for OSA is CPAP. CPAP has been shown to improve patients’ respiratory quality and quality of life, and may improve the prognosis of patients with pulmonary nodules to some extent. Studies have shown that long-term CPAP treatment is associated with a reduced incidence of lung cancer in OSA patients (HR = 0.79, 95%CI: 0.65–0.96) (64). CPAP can reduce the level of inflammation and oxidative stress in the airway of OSA patients, and may delay the progression of lung disease. Studies have shown that CPAP can significantly reduce the levels of inflammatory factors such as IL-6 and CRP in OSA patients, suggesting that it may inhibit pulmonary inflammation-related malignant transformation (65). CPAP improves vascular dysfunction and reduces oxidative stress in patients with metabolic syndrome and obstructive sleep apnea syndrome (66). The systematic review by Peker et al. (67) further confirms the improvement effect of CPAP on systemic inflammation.

### 5.2 Effect of treatment timing and adherence

CPAP can reduce HIF-1α and VEGF, which may inhibit the tumor microenvironment (68). Studies have shown that CPAP can regulate the expression of HIF-1α and VEGF in OSA patients, which has potential anti-tumor effect (69). However, adherence data from CPAP studies are often incomplete, which is an important factor affecting the assessment of treatment effect. Adherence to CPAP (duration of use per night) is a key factor in the effectiveness of treatment, but most studies do not report detailed data on adherence. Patients receiving CPAP tend to be a group with more severe symptoms, better compliance, and higher access to medical resources, which may themselves affect the detection and management of pulmonary nodules (70).

### 5.3 Multidisciplinary comprehensive management strategy

In the management of pulmonary nodules, the identification and treatment of OSA plays an important role. Therefore, early recognition and management of OSA is essential to improve the overall prognosis of patients with pulmonary nodules. OSA not only affects the prognosis of pulmonary nodules, but also may lead to a series of complications. Studies have shown that patients with OSA are more likely to have respiratory complications after surgery, especially in patients undergoing pulmonary surgery, and the presence of OSA significantly increases the risk of postoperative pulmonary complications (71).

Clinicians should consider performing a systematic evaluation of OSA when dealing with patients with pulmonary nodules, and make individualized treatment plans according to the specific conditions of patients. Early recognition and management of OSA is particularly important in patients with pulmonary nodules. The presence of OSA may aggravate the existing lung lesions and have a negative impact on the therapeutic effect. Therefore, clinicians should systematically screen for OSA when evaluating patients with pulmonary nodules, and formulate corresponding management strategies based on individual clinical characteristics. Such comprehensive management not only helps to improve the quality of life of patients, but may also reduce the risk of malignant transformation of pulmonary nodules.

## 6 Research limitations and future directions

### 6.1 Methodological limitations of the available evidence

Although existing studies suggest an association between OSA and pulmonary nodules and the risk of lung cancer, the current evidence is mainly derived from observational studies, which has important limitations in causal inference. According to the Grading of Recommendations Assessment Development and Evaluation (GRADE) system, the quality of evidence on the relationship between OSA and pulmonary nodules is generally low (72–74).

An inherent limitation of observational studies is the inability to establish causality. The observed association may be explained by:

1) OSA does lead to an increased risk of pulmonary nodules (causal effect);
2) OSA caused by pulmonary nodules or related factors (reverse causality);
3) There are unmeasured confounding factors affecting both OSA and pulmonary nodules (confounding bias);
4) Pure chance (random error) (75, 76). A number of key studies were retrospective in design, and there were methodological problems such as selection bias, information bias and recall bias.

### 6.2 The challenge of controlling confounding factors

There are obvious deficiencies in the control of confounding factors in the existing studies. Smoking is the strongest risk factor for lung cancer and pulmonary nodules, and it is also an important related factor for OSA. Smokers have a significantly higher prevalence of OSA than non-smokers, which may be related to airway inflammation and upper airway neuromuscular dysfunction caused by smoking (77, 78). However, a number of key studies have not adequately controlled for the confounding factor of smoking.

Obesity is an independent risk factor for OSA, and obesity-related chronic inflammation may also affect the risk of lung cancer. Existing studies have problems in BMI control. Some studies only adjusted BMI as a categorical variable, which may not capture the nonlinear relationship between BMI and outcomes. BMI cannot fully reflect fat distribution, and abdominal obesity may be more closely related to OSA than overall obesity (79). In addition, factors such as occupational exposure, air pollution exposure, family history, and comorbidities are often not fully considered in existing studies (Figure 1).

### 6.3 Priority areas for future research

Based on the above analysis, future research should focus on the following priority scientific questions, and actively explore the translational application of emerging technologies in clinical practice.

#### 6.3.1 Priority research questions

##### 6.3.1.1 Randomized controlled trials (RCTS) of CPAP treatment

The current evidence mainly comes from observational studies. Whether CPAP treatment can really reduce the growth rate and malignant transformation rate of pulmonary nodules is still lack of high-quality RCTS verification. Future multicenter, prospective RCTS should be designed to definitively answer the following question: Does CPAP therapy slow the volume growth of established pulmonary nodules? Can the malignant transformation rate of nodules be reduced? What are the optimal treatment timing and adherence thresholds? Such studies will provide level I evidence for the clinical application of CPAP in the management of pulmonary nodules (80).

##### 6.3.1.2 The effect of OSA screening on the prognosis of lung cancer

Can systematic screening for OSA in high-risk groups of lung cancer (such as long-term smokers and people with family history) improve the detection rate of early lung cancer and the survival prognosis of patients? Prospective cohort studies are needed to evaluate the synergistic effect of OSA screening combined with low-dose CT (LDCT) and to clarify the risk stratification value of OSA as an independent risk factor for lung cancer (79).

##### 6.3.1.3 The dose–response relationship of intermittent hypoxia

Is there a clear dose–response relationship between OSA severity (as measured by AHI and the lowest oxygen saturation at night) and the risk of pulmonary nodule malignancy? The identification of critical thresholds is critical for risk stratification strategies and clinical decision making.

##### 6.3.1.4 Clinical validation of the biological mechanism

Can laboratory indicators such as HIF-1α/NF-κB pathway, oxidative stress markers, and inflammatory factors be used as predictive biomarkers for the risk of OSA-related pulmonary nodules? The actual predictive value of these mechanistic pathways needs to be verified in clinical samples.

#### 6.3.2 Clinical translation of emerging tools

##### 6.3.2.1 Artificial intelligence (AI) assisted diagnosis

Deep learning algorithms have shown excellent performance in the detection of pulmonary nodules and the discrimination of benign and malignant nodules. Future research should develop AI prediction models that integrate OSA clinical features (such as AHI and nocturnal hypoxia indicators) to improve the accuracy of individualized assessment of the malignant risk of pulmonary nodules in OSA patients. Ai-assisted decision systems can help clinicians identify high-risk nodules that require intensive follow-up and optimize resource allocation (62, 63).

##### 6.3.2.2 Multiple groups of student biomarkers

A multi-omics strategy based on genomics, proteomics and metabolomics is expected to find specific biomarkers for OSA-related pulmonary nodules. Liquid biopsy techniques such as circulating tumor DNA (ctDNA), exosomal miRNA, and inflammatory factor profiling may realize non-invasive risk stratification and early warning. In the future, a multimodal prediction model integrating clinical-radiomics data should be established (81).

##### 6.3.2.3 Wearable devices and remote monitoring

The development of portable sleep monitoring devices and smart wearable technology has made large-scale screening and long-term follow-up of OSA more convenient. Combined with the remote pulmonary nodule imaging follow-up system, a whole-process management model for patients with OSA and pulmonary nodule comorbidity can be established to improve the compliance of diagnosis and treatment and prognosis (15).

#### 6.3.3 Suggestions for study design optimization

Future studies should improve the methodological quality in the following aspects:

1) Conducting large-scale prospective cohort studies with nested case–control design to improve efficiency;
2) Exploring Mendelian randomization studies using genetic variants as instrumental variables to infer causality;
3) Strengthening the control of confounding factors: smoking history (quantified by pack-year), abdominal obesity indicators such as waist circumference/waist-hip ratio, occupational and environmental exposure history were collected in detail;
4) Standardized diagnosis and outcome evaluation: unified use of polysomnography to diagnose OSA, establish a standardized scheme for imaging evaluation of pulmonary nodules, and distinguish different pathological types of pulmonary nodules (82).

## 7 Conclusion

In conclusion, the relationship between OSA and pulmonary nodules has important clinical implications. Current epidemiological evidence suggests that patients with OSA have an increased incidence of pulmonary nodules, and the severity of OSA is associated with the risk of malignant nodules. Intermittent hypoxia activates oxidative stress and inflammatory response through HIF-1α/NF-κB signaling pathway, which may be the core biological mechanism of OSA promoting the occurrence and development of pulmonary nodules. CPAP treatment can reduce the levels of inflammatory factors and may delay the malignant transformation of pulmonary nodules, which provides a potential target for clinical intervention.

However, limited by observational study design, insufficient control of confounding factors, inconsistent diagnostic criteria, and other problems, the current evidence is not enough to establish the causal relationship between OSA and pulmonary nodules. Clinicians should be cautious when assessing the risk of pulmonary nodules in OSA patients, comprehensively consider multiple risk factors, and avoid over-interpretation of the existing evidence. In the future, higher quality studies are needed to clarify the true relationship between OSA and pulmonary nodules and its potential mechanism, so as to provide a more solid theoretical basis for the formulation of clinical preventive measures.
